# Diverse Gastropod Hosts of *Angiostrongylus cantonensis*, the Rat Lungworm, Globally and with a Focus on the Hawaiian Islands

**DOI:** 10.1371/journal.pone.0094969

**Published:** 2014-05-02

**Authors:** Jaynee R. Kim, Kenneth A. Hayes, Norine W. Yeung, Robert H. Cowie

**Affiliations:** 1 Department of Biology, University of Hawaii, Honolulu, Hawaii, United States of America; 2 Pacific Biosciences Research Center, University of Hawaii, Honolulu, Hawaii, United States of America; 3 Department of Biology, Howard University, Washington, District of Columbia, United States of America; INSERM U1094, University of Limoges School of Medicine, France

## Abstract

Eosinophilic meningitis caused by the parasitic nematode *Angiostrongylus cantonensis* is an emerging infectious disease with recent outbreaks primarily in tropical and subtropical locations around the world, including Hawaii. Humans contract the disease primarily through ingestion of infected gastropods, the intermediate hosts of *Angiostrongylus cantonensis*. Effective prevention of the disease and control of the spread of the parasite require a thorough understanding of the parasite's hosts, including their distributions, as well as the human and environmental factors that contribute to transmission. The aim of this study was to screen a large cross section of gastropod species throughout the main Hawaiian Islands to determine which act as hosts of *Angiostrongylus cantonensis* and to assess the parasite loads in these species. Molecular screening of 7 native and 30 non-native gastropod species revealed the presence of the parasite in 16 species (2 native, 14 non-native). Four of the species tested are newly recorded hosts, two species introduced to Hawaii (*Oxychilus alliarius*, *Cyclotropis* sp.) and two native species (*Philonesia* sp., *Tornatellides* sp.). Those species testing positive were from a wide diversity of heterobranch taxa as well as two distantly related caenogastropod taxa. Review of the global literature showed that many gastropod species from 34 additional families can also act as hosts. There was a wide range of parasite loads among and within species, with an estimated maximum of 2.8 million larvae in one individual of *Laevicaulis alte*. This knowledge of the intermediate host range of *Angiostrongylus cantonensis* and the range of parasite loads will permit more focused efforts to detect, monitor and control the most important hosts, thereby improving disease prevention in Hawaii as well as globally.

## Introduction

*Angiostrongylus cantonensis* is a parasitic nematode and one of the major causes of eosinophilic meningitis, a potentially fatal disease in humans and other mammals, as well as birds [1]–[6]. Additional causes of eosinophilic meningitis include other parasitic, bacterial, viral and fungal infections, as well as intracranial malignancies or medical devices and allergic reactions to drugs [7]. *Angiostrongylus cantonensis* has been recorded on all continents except Europe and Antarctica and over 2,800 human cases of eosinophilic meningitis caused by it have been reported from about 30 countries [8], [9]. Most records of the disease, also known as rat lungworm disease, have been from tropical and subtropical areas in Southeast Asia and the Pacific Basin. However, cases have also been sporadically reported in other regions, including places where *A. cantonensis* is not present, when people return from regions where it occurs [8]–[13].

Definitive hosts of *A. cantonensis* include various rat species, mainly in the genus *Rattus*, which become infected by ingesting intermediate hosts (gastropods) or paratenic hosts (e.g. frogs, crabs, prawns, planarians) containing third stage *A. cantonensis* larvae [14]–[18]. These larvae mature fully and reproduce in the rat, resulting in eggs that hatch into first stage larvae. The first stage larvae are ultimately released in the rat's feces [9], [19], which may then be ingested by the gastropod intermediate hosts. The ingested first stage larvae go through two molts to become third stage larvae while in the intermediate host, which is then consumed by the definitive host and the cycle repeats [19].

Numerous birds and mammals, including humans, are accidental hosts and are infected in the same manner as rats [3]–[5]. However, in these accidental hosts the larvae die when they reach the central nervous system, primarily in the brain, which can lead to eosinophilic meningitis [20]. In humans, the resulting symptoms include nausea and headache, and in more severe cases, neurologic dysfunction, coma, and death [8], [21]. The severity of the symptoms depends on the parasite load of the infected gastropod ingested, which can vary within and among snail species [15], [21]–[26]. Ingestion of infective larvae can be as a result of either deliberate or accidental ingestion of infected intermediate hosts [27]–[29]. In other mammals, as well as birds, there are also severe neurological manifestations, including mortality [3]–[5].

The spread of *A. cantonensis* has been driven by human activity, through dispersal of definitive and intermediate hosts. Definitive hosts have long been associated with human travel and trade and if infected provide a source of *A. cantonensis* in areas where snails occur [8], [30]. Snail intermediate hosts are also easily dispersed by human activities, and are transported around the world both intentionally and accidentally by various pathways, notably the agricultural and horticultural industries [31], [32]. As a result of the increased movement of these hosts around the world, eosinophilic meningitis caused by *A. cantonensis* is an emerging infectious disease, increasing in incidence and expanding in geographical range [7], [33]. With global climate change, suitable habitat for intermediate hosts may increase and regions with appropriate conditions for parasite transmission to occur could expand. Thus, *A. cantonensis* may expand from being only a tropical concern to a more global one [34], [35].

Since the first reported cases in the Hawaiian Islands in 1960 [36], human infection by *A. cantonensis* has become increasingly prevalent there. From 2001 to 2012 there have been approximately 60 reported cases ([37]–[39], S. Y. Park, personal communication, November 2013). Most cases were probably caused by infection following accidental consumption of live gastropods and the consumption of produce containing infected gastropods [28].

In Hawaii, there are more than 750 recognized native land snail species, a similar number to the fauna of the continental United States and Canada combined. However a high proportion of these native species are now extinct, with most of those remaining now confined to high elevation refugia away from human disturbance [40]–[43]. The number of established non-native gastropod species in the Hawaiian Islands (43) is also the highest among the islands of the Pacific ([32], [44]–[46], Hayes et al. unpublished). Because snails are the intermediate hosts of *A. cantonensis*, determining which snail species carry the parasite is important for understanding the geographical spread of the disease, both on a global scale and locally from the perspective of public health management in the Hawaiian Islands. This study focused on the non-native species because these are the snails that are being transported around the world [32], [47] and are the ones likely to be spreading the disease. They may also be involved in passing the parasite on to the remaining, severely threatened native taxa, creating an additional concern from disease ecology and snail conservation perspectives as the impacts of the parasites on the snails is unknown. Therefore, the aim of this study was to screen a large cross section of the non-native gastropod species throughout the Hawaiian Islands, as well as a smaller representative number of the native species, to determine which of them act as intermediate hosts of *A. cantonensis*. A secondary aim was to assess the parasite loads in these species to identify those with the greatest potential for infecting people. We achieved this by screening non-native species from 12 out of the 30 families present in Hawaii, as well as native species from two of a total of ten families.

## Materials and Methods

### Ethics Statement

Field studies did not involve endangered or protected species. Collecting permits for all state and federal lands surveyed were provided by the Department of Land and Natural Resources and Oahu Army Natural Resources Program. Surveys in nurseries and other horticultural facilities were done with the permission of the owners.

### Sampling and specimen selection

**Figure 1:**
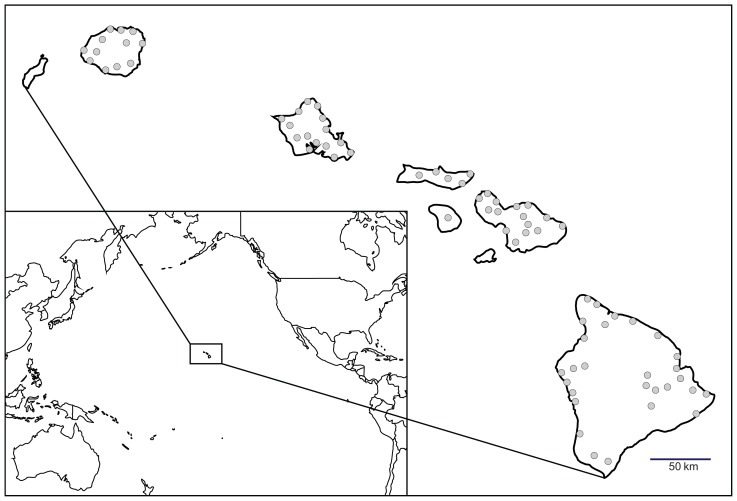
The extent of gastropod sampling throughout the main Hawaiian Islands. To show the broad geographic coverage, the map includes only sites 10 km or more away from each other.

Between 2004 and 2013, surveys were conducted on the six largest Hawaiian Islands to determine the distributions of non-native and native gastropods ([32], [46], [48], unpublished data). Field studies did not involve endangered or protected species. Over 9,500 live specimens (ca 250+ species, including many undescribed native species) were collected during these surveys and preserved in 75% and 95% ethanol for morphological and molecular work, respectively. Non-native (n = 1,062) and native (n = 209) gastropod specimens were selected from these collections for screening to provide a broad coverage of species (37 species, 30 of them non-native and 7 native) and locations (182 sites) (Figure 1), including in particular species previously recorded as carriers of *A. cantonensis* and those known to be widespread throughout the main Hawaiian Islands according to Cowie et al. [32]. Collecting permits for all state and federal lands surveyed were provided by the Department of Land and Natural Resources and Oahu Army Natural Resources Program. Surveys in nurseries and other horticultural facilities were done with the permission of the owners.

### Molecular detection of *A. cantonensis*

The larvae of *A. cantonensis* are distributed throughout the host gastropod's body, although differentially among the various organs [49], [50], and encyst in the host tissue [51]. To extract nematode DNA, total genomic DNA was isolated from ca 0.2 mg of foot tissue for the smallest snails up to ca 10 mg for the largest. Extractions were carried out using the IDPure Spin Column Plant Genomic DNA Isolation Kit following the manufacturer's instructions.

Initial tests to determine if the snails served as hosts for the nematode were done by amplifying a ca 1,134 bp fragment of 18S rDNA sequence using primers (AngioF1 and AngioR1) specific to the superfamily, Metastrongyloidea, to which *A. cantonensis* belongs following Qvarnstrom et al. [52]. Amplifications were performed in 25 µl reactions with a final concentration of 1X reaction buffer (ID Labs, London, ON, Canada), 0.2 mM of each dNTP, 2 mM MgCl_2_, 1.25 U of IDPROOF DNA polymerase (ID Labs, London, ON, Canada), 0.16 µM of each primer, 0.4 µg/µl of BSA, 0.5% DMSO and 2 µl of template DNA. A touchdown protocol was used to promote specific amplification of *A. cantonensis* DNA from total gastropod DNA extracts. Amplification parameters were 95°C for 5 min, 7 cycles of 95°C for 20 s, 65°C for 20 s with a 1°C decrease per cycle, and elongation at 72°C for 45 s followed by 35 cycles of 95°C for 20 s, 59°C for 20 s, and 72°C for 45 s, and a final elongation at 72°C for 10 min. Reactions were terminated with a 4°C hold for 30 min. Amplified fragments were visualized on an agarose gel to confirm size and quality. All reaction sets included a negative control and positive controls verified by previous amplifications. To evaluate the specificity of amplifications, 30% of the positive amplicons were cleaned using the IDPure Purification Kit (ID Labs, London, ON, Canada) and sequenced with the forward primer (AngioF1) at the Greenwood Molecular Biology Facility (Pacific Biosciences Research Center, University of Hawaii). The sequences were compared with known *A. cantonensis* sequences [53], [54].

To verify that positives from the 18S PCR assay were the result of infection by *A. cantonensis*, all positive samples and a random subset (ca 10%) of the negative samples were retested with a PCR assay aimed at amplifying the ribosomal internal transcribed spacer one (ITS1) region using the species-specific primers AcanITS1F1 and AcanITS1R1, following Qvarnstrom et al. [55]. These primers can detect false negatives that are missed with 18S, and reveal false positives resulting from non-specific amplification of other species of Metastrongyloidea [55]. Amplifications were performed in 25 µl reactions as for 18S with the exception of using 3 µl of template DNA. Amplification parameters were 95°C for 3 min, 45°C for 1 min, and elongation at 72°C for 1 min followed by 35 cycles of 95°C for 20 s, 48°C for 20 s, and 72°C for 35 sec with a final elongation at 72°C for 10 min. Amplicons were visualized as for 18S.

### Quantification of parasite load

The parasite loads of all specimens that tested positive for *A. cantonensis* were quantified following the real-time, quantitative PCR (RT-PCR) TaqMan assay of Qvarnstrom et al. [55]. First, a standard curve was generated from DNA extractions containing an estimated 1.744–1,744 larvae. To do this, nematodes were isolated from tissue of a live *Parmarion martensi* that was known to be infected, using a modification of the protocol of Wallace and Rosen [56] with a higher pepsin concentration (S. C. Thiengo, personal communication to K. A. Hayes, November 2008). The specimen was minced, put in 50 ml of digestion solution containing 3% pepsin and 0.7% HCl and incubated at room temperature with occasional agitation. The solution was then transferred to a Baermann apparatus with a stoppered tube attached to the bottom of the funnel containing a 2×2 cm piece of medical gauze. The solution containing nematode larvae was left overnight to allow the larvae to migrate through the gauze and into the stoppered tube, after which the solution was recovered by draining the solution from the tube into a 50 ml beaker. Larvae, which remain alive and intact following digestion, were concentrated into 3 ml of solution from which five 100 µl samples were taken. The number of larvae in each 100 µl sample was counted under a stereomicroscope and the average concentration of the five samples was used to determine the total number of nematode larvae per 100 µl. Based on this estimated concentration of larvae, five samples were drawn from the remaining 2.5 ml of solution containing 1.744 (1 µl), 8.72 (5 µl), 17.44 (10 µl), 174.4 (100 µl), and 1,744 (1 ml) larvae. From each of these samples, DNA was extracted as described previously and eluted in 80 µl of elution buffer. Because of the possibility of co-isolated products inhibiting PCR all samples were diluted 1∶5 and then amplified in triplicate. Cycling conditions differed slightly from those of Qvarnstrom et al. [55], starting with 2 min at 50°C, 2 min at 90°C, and finishing with 40 cycles of 15 sec at 95°C and 1 min at 60°C. All RT-PCR assays were carried out at the State of Hawaii Department of Health using an Applied Biosystems 7500 Fast Real-time PCR System v1.4.0 and analyzed using Applied Biosystems 7500 Fast System with 21 CFR Part 11 software.

A standard curve was generated by plotting the cycle threshold (C_T_) values obtained from the RT-PCR of estimated quantities of parasites against the log number of estimated parasites in those samples, permitting generation of a linear equation that was used to estimate the number of larvae in a sample from its C_T_ value. To estimate the number of larvae in each specimen, the ethanol preserved snails were patted dry in tissue paper for one minute and weighed. Shells were removed from larger snails, but for small snails the combined shell and body weight was used. The average weight of tissue used for DNA extraction of each species was also estimated by sampling a piece of tissue from five similarly sized specimens, patting them dry for one minute and weighing them. The total number of larvae was then estimated for each specimen by extrapolation.

### Statistical analyses

Statistical analyses were carried out using Microsoft Excel 2013. Rates of infection of non-native and native species were compared using the chi-square test. This test was also used to assess differences in rates of infection in species with different habits as assessed in the field (primarily ground-dwelling, arboreal, freshwater, both ground-dwelling and arboreal), as ground-dwelling snails may have increased chances of encountering rat feces compared to arboreal and aquatic snails. Differences between means were considered statistically significant if the P-value was less than 0.05.

## Results

**Figure 2:**
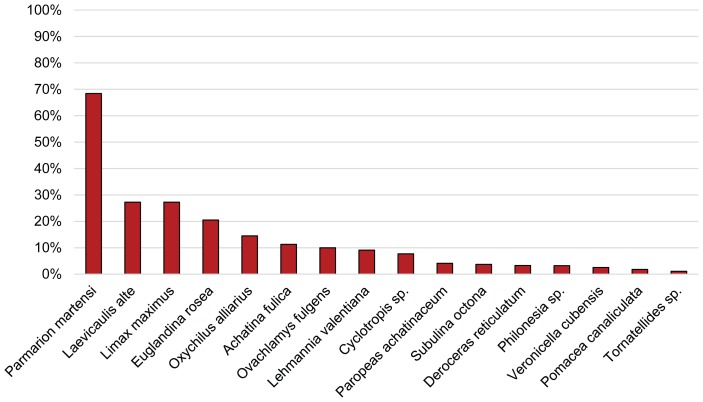
Infection rates for gastropod species that tested positive in this study. Levels of infection vary considerably from 68% infection in *Parmarion martensi* to 1% in *Tornatellides* sp.

The 18S and ITS1 amplifications gave identical results. Of the 37 species, 16 tested positive for *A. cantonensis*, with 70 specimens testing positive out of a total of 1,271 (Table 1). Among the 30 non-native species, 14 tested positive, two being newly recorded natural hosts of *A. cantonensis* (*Cyclotropis* sp., *Oxychilus alliarius*). Of a total of 1,062 non-native gastropods, 6% were positive for *A. cantonensis*. No specimens of four non-native species (*Bradybaena similaris*, *Deroceras laeve*, *Limax flavus*, *Melanoides tuberculata*) that have been recorded in other studies as hosts of *A. cantonensis* (Appendix S1) tested positive for the parasite. *Parmarion martensi* had the highest prevalence of infection with 68% (13/19) of the specimens testing positive for *A. cantonensis* followed by *Laevicaulis alte* with an infection rate of 30% (13/44) (Table 1, Figure 2).

**Table 1:** Infection rates and average parasite loads (of positive specimens) in the gastropod species screened in this study.

Family	Species	Habit	No. tested	No. (%) positive	No. sites positive (total)	Average C_T_ 1∶5 dilution value (range)	Average no. of parasites per 5 mg of snail tissue	Average no. of parasites in entire specimen (range)
**Achatinellidae**	*Auriculella* spp.	A	31	0	0 (8)	-	-	-
	*Elasmias* spp.	B	18	0	0 (9)	-	-	-
	*Lamellidea* spp.	B	25	0	0 (13)	-	-	-
	*Tornatellides* spp.	B	90	1 (1)	1 (30)	− (24.66)	-	− (2,379)
**Achatinidae**	Achatina fulica*	G	62	7 (11)	4 (21)	25.66 (20.16–31.26)	237	213,515 (14,379–870,868)
**Agriolimacidae**	Deroceras laeve*	G	79	0	0 (27)	-	-	-
	Deroceras reticulatum*	G	61	2 (3)	1 (23)	25.24 (23.54–26.94)	1,564	9,789 (3,500–16,078)
**Ampullariidae**	Pomacea canaliculata*	F	56	1 (2)	1 (15)	− (23.78)	-	− (68,133)
**Arionidae**	*Arion intermedius*	G	20	0	0 (8)	-	-	-
	*Arion subfuscus*	G	8	0	0 (4)	-	-	-
**Ariophantidae**	Parmarion martensi*	G	19	13 (68)	5 (8)	24.23 (17.28–29.43)	912	55,852 (850–341,828)
**Assimineidae**	*Cyclotropis* sp.	G	13	1 (8)	1 (3)	− (28.60)	-	− (154)
**Bradybaenidae**	Bradybaena similaris*	G	65	0	0 (16)	-	-	-
**Euconulidae**	*Liardetia doliolum*	G	8	0	0 (5)	-	-	-
**Gastrodontidae**	*Zonitoides arboreus*	G	18	0	0 (5)	-	-	-
**Helicarionidae**	*Kaala subrutila*	G	2	0	0 (1)	-	-	-
	Ovachlamys fulgens*	G	10	1 (10)	1 (4)	− (22.42)	-	− (11,118)
	*Philonesia* sp.	A	31	1 (3)	1 (11)	− (24.57)	-	− (4,823)
**Helicidae**	*Cornu aspersum*	G	25	0	0 (8)	-	-	-
**Limacidae**	Lehmannia valentiana*	G	11	1 (9)	1 (6)	− (22.19)	-	− (24,819)
	Limax flavus*	G	8	0	0 (4)	-	-	-
	Limax maximus*	G	11	3 (27)	2 (4)	22.22 (20.36–24.35)	1,960	398,160 (170,067–566,582)
**Lymnaeidae**	*Fossaria viridis*	F	18	0	0 (4)	-	-	-
**Milacidae**	*Milax gagates*	G	22	0	0 (7)	-	-	-
**Orthalicidae**	*Bulimulus guadalupensis*	G	10	0	0 (2)	-	-	-
**Oxychilidae**	*Oxychilus alliarius*	G	69	10 (14)	6 (17)	25.69 (20.22–34.41)	1,922	13,382 (63–55,807)
**Planorbidae**	*Planorbella duryi*	F	20	0	0 (6)	-	-	-
**Physidae**	*Physa* spp.	F	27	0	0 (6)	-	-	-
**Spiraxidae**	Euglandina rosea*	G	39	8 (21)	5 (16)	27.66 (24.37–33.13)	166	43,687 (1,244–113,645)
**Streptaxidae**	*Gonaxis kibweziensis*	G	11	0	0 (5)	-	-	-
**Subulinidae**	Paropeas achatinaceum*	G	73	3 (4)	2 (18)	27.16 (25.77–29.36)	1,518	11,421 (1,724–21,087)
	Subulina octona*	G	54	2 (4)	1 (13)	24.60 (23.21–25.99)	3,548	39,114 (15,835–62,392)
**Succineidae**	*Succinea caduca*	G	12	0	0 (4)	-	-	-
	*Succinea tenella*	G	25	0	0 (6)	-	-	-
**Thiaridae**	Melanoides tuberculata*	F	17	0	0 (5)	-	-	-
**Veronicellidae**	Laevicaulis alte*	G	44	13 (30)	11 (21)	24.99 (17.32–31.42)	1,592	342,971 (4,127–2,801,566)
	Veronicella cubensis*	G	159	4 (3)	3 (45)	23.40 (21.35–24.93)	531	116,891 (28,931–253,909)
**Total**			**1271**	**71 (6)**	**40 (182)**			

1 Previously recorded as a host in the Hawaiian Islands and/or elsewhere.

2 Native Hawaiian gastropod species. Habits are ground-dwelling (G), arboreal (A), both ground-dwelling and arboreal (B) and freshwater (F).

Of the seven native Hawaiian species screened, two tested positive (one individual of each of *Philonesia* sp. and *Tornatellides* sp.). The proportion of native snails found to carry *A. cantonensis* was significantly smaller than the proportion of non-native snails that tested positive (χ^2^ = 9.95, df = 1, P = 0.002). The proportions of all snails infected were significantly related to their habit (χ^2^ = 18.5, df = 3, P = 0.0004): 7% (67/938) of the individuals of ground-dwelling species tested positive; arboreal and freshwater snails were less susceptible at 2% (1/62) and 0.7% (1/138), respectively; and 0.8% (1/133) of the individuals of species that are both ground-dwelling and arboreal tested positive. Of 182 sites from which snails were screened, 40 had snails that tested positive for *A. cantonensis* (Table 1). Infected *L*. *alte* were found at more sites (11) than any other species.

The linear equation derived from RT-PCR standard curve data was 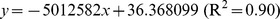
where y is the C_T_ value and x is the number of larvae in the sample. The numbers of parasites in each DNA extraction were estimated using this equation and ranged from 2 to 6,427. Extrapolating from these data to the intensity of infection (number in 5 mg of tissue) and the total number in each individual, showed that parasite load varied widely both within and among species (Table 1). *Subulina octona* had the highest average concentration of parasites (3,548 per 5 mg tissue). However, a *L. alte* specimen had the highest individual parasite concentration (8,147 parasites per 5 mg of tissue), followed by an *O. alliarius* (7,068) and a *P. martensi* (6,639). The two caenogastropod species, *Pomacea canaliculata* and *Cyclotropis* sp. had the lowest average parasite concentrations, more than 20 times less than that in *S. octona*, while an *A. fulica* had the lowest individual parasite concentration at 6 parasites per 5 mg of tissue. Average total parasite loads were highest in *L. maximus*, *L. alte* and *A. fulica* (398,160, 342,971 and 213,515, respectively) and individuals of these species also had the highest individual loads (566,582, 2,801,566 and 870,867, respectively). These high numbers are based on significant extrapolation beyond the standard curve. Theoretically the curve should be linear but at such high levels of infection it may not be and these numbers may be over-estimates. Also, the concentration of larvae may differ among the various parts of the snails' bodies [49], [50]. (Such caveats are also applicable to studies that extrapolated from samples in which larvae were counted visually.) The species with the lowest average parasite load was *Cyclotropis* sp. (154 parasites), but an *O. alliarius* specimen had the lowest individual load (63).

**Figure 3:**
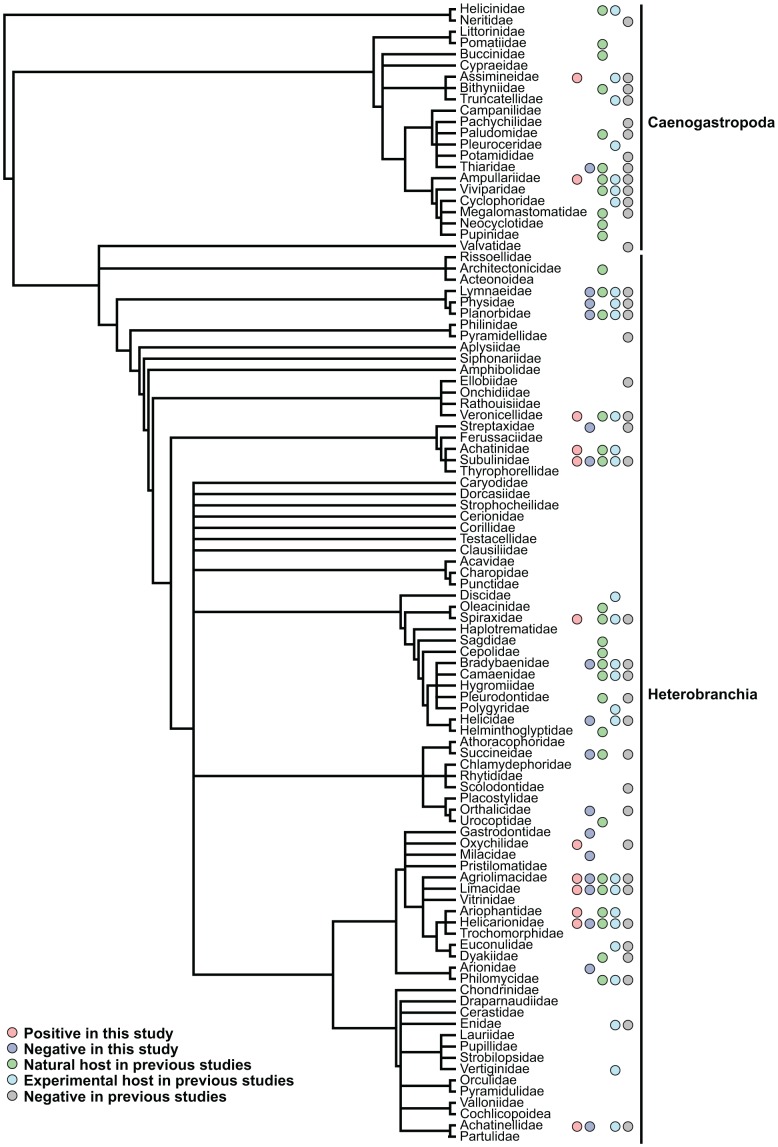
Phylogeny of mollusc families showing which have been recorded as intermediate hosts of *Angiostrongylus cantonensis*. Phylogeny constructed using the classifications and phylogenies of Bouchet and Rocroi, Aktipis et al. and Strong et al. [59]–[61], indicating the diversity of families in which mollusc species have been shown to act as hosts of *Angiostrongylus cantonensis*. Bars at the right of the tree indicate the taxonomic group that the families belong to.

We have now shown that nearly a third of the non-native snail species established in the Hawaiian Islands are carriers of *A. cantonensis* along with two native species (*Philonesia* sp., *Tornatellides* sp.). Others may also act as carriers but were just not recorded as such in this study. In addition, many other species act as hosts in other parts of the world (Appendix S1). Including the current study, species from 58 families (out of 409 extant gastropod families) [59] have now been evaluated for their potential to carry *A. cantonensis*, and all but 12 of these families contained taxa that were capable of carrying the nematode (Figure 3).

## Discussion

The diversity of gastropods now known to carry *Angiostrongylus cantonensis* encompasses a broad phylogenetic range of both terrestrial and freshwater species (Figure 3; Appendix S1). A single fully marine species (*Discotectonica acutissima*: Architectonicidae) has also been reported as testing positive for *A. cantonensis* [57] but this is a sublittoral (50–200 m depth) species [58] and the finding may be incorrect. Previous studies had identified species in 33 families as natural hosts of *A. cantonensis*, with the present study adding three more: Achatinellidae, Assimineidae and Oxychilidae. Species in another 10 families have been shown experimentally to be capable of acting as hosts. Individuals from 12 families were positive for *A. cantonensis* in the current study. These included not only highly divergent heterobranchs (which includes approximately 80% of all land snails) such as *Tornatellides* sp. (Achatinellidae), *V. cubensis* (Veronicellidae) and *P. martensi* (Ariophantidae) but also caenogastropods (the largest gastropod group, mostly marine), i.e. *Cyclotropis* sp. and *P. canaliculata*. Based on dietary habits, detritivores, which more often come in contact with rat feces, might be anticipated to have a higher incidence of infection. However, herbivorous species such as *Veronicella cubensis* [62], [63], predatory species like *Euglandina rosea* [64], and detritivores like *S. octona* [65], all tested positive, despite their dietary differences. Although the molecular approach used in this study was not able to distinguish larval stages of the parasite and therefore whether the parasite can develop to the infective stage in all species that tested positive, the majority of studies listed in Appendix S1 were morphological and detected third stage larvae. Therefore, the extremely broad diversity of gastropods in which *A. cantonensis* has been found, indicates that almost any terrestrial or freshwater gastropod may have the potential to carry and transmit the parasite, which has broad implications for its continued spread.

Two families (Achatinidae, Ariophantidae) exhibited a high infection rate, inasmuch as they included species that were reported as natural hosts and/or had been successfully infected experimentally in this and all previous studies that screened them for *A. cantonensis*. Species in two families (Orthalicidae and Streptaxidae) tested negative in this and all previous studies, perhaps because they have low susceptibility to infection or the individuals tested came from localities where the frequency of infection of intermediate hosts was low. Ten families were represented by species that were only reported as having been infected experimentally, including the Truncatellidae, which live close to the sea shore where the probability of transmission may be low because wave action washes rat feces away.

As predicted, ground-dwelling species tested positive for *A. cantonensis* more frequently than arboreal and freshwater species, probably because of their more ready access to rat feces. Four freshwater species (*Fossaria viridis*, *Melanoides tuberculata*, *Planorbella duryi*, *Physa* sp.) tested negative for *A. cantonensis* in this study and only 1 of 56 (2%) *P. canaliculata* tested positive. It may be more difficult for snails in freshwater habitats to acquire the parasite as access to rat feces in streams and rivers may be limited [66]. However, other studies have identified a number of freshwater species able to carry *A. cantonensis*, including *P. canaliculata* and *M. tuberculata* (Appendix S1). The rate of infection of *M. tuberculata* was often less than 1%, whereas *P. canaliculata* showed higher rates of up to 40% [23], [67]–[71]. The low infection rate in *P. canaliculata* in the present study could be due to the majority of these specimens being from irrigated areas such as taro patches, some with flowing water, where incidence of infection may be low because of lower concentrations of parasites in the water than in intact rat feces in terrestrial situations [72]. In Asia, *P. canaliculata* is commonly identified as the source of infection in human cases of angiostrongyliasis, not necessarily because of the high percentage of infected snails, but because of its popularity as a food source [73], [74].

A number of terrestrial snails also did not test positive for *A. cantonensis*. For instance, none of the 65 *Bradybaena similaris* specimens tested positive in this study. It is possible that *B. similaris* is naturally less susceptible to *A. cantonensis* infection than other species, as in a previous study only 8 of 281 (4%) *B. similaris* specimens from Oahu were infected [15]. All *Arion intermedius*, *Arion subfuscus* and *Cornu aspersum* specimens tested were from sites higher than 600 m above sea level, where the parasite may not yet be at high densities or may be limited by temperature. Alternatively, at least for *C. aspersum*, nematode inhibitors that prevent maturation or reproduction and that have been isolated from this species [76], [77], may explain its low infection rate. In another study, no parasites were recovered from several hundred *C. aspersum* in New Caledonia [78], which lends support to this possibility.

*Achatina fulica* is well known as an intermediate host of *A. cantonensis* (e.g. [11], [74], [79], [80]). However, the level of infection of *A. fulica* varies widely among localities. In the present study only 7 of 62 (11%) *A. fulica* tested positive. In one study in Brazil, Neuhauss et al. [81] found only one infected *A. fulica* out of 244 (0.4%) screened, while in another Thiengo et al. [26] found 14 among 33 (42%) screened. The level of infection at different locations in Guangdong, China, varied widely from 0 to 45.4% [82]. The most likely explanation for this variability may be the variation in presence and abundance of *A. cantonensis* in different environments, possibly related to abiotic factors such as temperature and humidity, but perhaps also to the distribution of infected rats, the species of rats present or differences in the interactions between rats and gastropods.

Among the newly recorded hosts, *Oxychilus alliarius* is a widespread European species. While *A. cantonensis* is primarily a tropical and subtropical parasite, presumably because it is constrained by ambient temperatures (which determine the temperature of its poikilothermic gastropod intermediate hosts), the fact that it can infect temperate gastropod species indicates that global warming trends may allow it to establish more widely in locations where such hosts are already present.

This is the first report of native Hawaiian snails carrying *A. cantonensis*. Most extant native snails are confined to high elevation habitat and rarely encountered by people. They are therefore unlikely to be important in transmission of *A. cantonensis* to humans. However, this finding may have negative implications for the health of the native Hawaiian snail fauna, which is especially vulnerable to additional threats. Once consisting of over 750 species [42], the fauna has declined drastically and is being replaced by a much smaller number of alien species [32], [43], [46], [48]. The deliberate introduction of predatory snails, notably *Euglandina rosea*, for use as biocontrol agents in ill-conceived efforts to control *Achatina fulica* has had a devastating effect on the native snails [43], [44], [83]. Habitat destruction has also been of major significance in the decline of the native fauna [84]. The snail fauna may be facing an additional threat if infection with *A. cantonensis* reduces the snails' fitness. Native snails are important in the functioning of healthy ecosystems and the possible impacts on the native fauna could have serious implications for ecosystem health. Native snails in Jamaica also carry *A. cantonensis*, but little is known about the effect of the parasite on their fitness [13], [85]. Richards and Merritt [51] recorded a clear tissue reaction to the parasite in *Biomphalaria glabrata*. This type of response to parasitic infection may be costly, using energy usually allocated towards survival and reproduction [86], [87]. Wallace and Rosen [75] showed that mortality was higher in *Physa elliptica* experimentally infected with *A. cantonensis* than in uninfected controls.

Although only 2 out of 210 specimens of native species tested positive for the parasite, this does indicate that the parasite may now have become sufficiently widespread and abundant to begin to infect native snail populations. Most native Hawaiian snails are very sparsely distributed, mostly at high elevations where *A. cantonensis* may not be able to survive in rat feces or develop in the snails. Nonetheless, these high elevation refugia generally support more than one species of native snail, so native species other than those testing positive in this study may yet be found to be susceptible to infection.

Susceptibility to infection may be related to the snails' behavior, location or physiology [78]. For example, *P. martensi*, a species that is mainly ground-dwelling is highly susceptible, with 68% of the specimens in this study testing positive, and often heavily infected [50], [52], [55], [88], [89]. However it will readily climb and is often found in trash cans and compost piles, where contact with rats and rat feces is probably a common occurrence [89]. These factors may also affect parasite load, which differed greatly within and among species. One *P. martensi* specimen had one of the highest parasite concentrations in its tissue and had on average as many larvae in its entire body as other species four to six times its mass (Table 1). Jarvi et al. [50] also showed that this species can support high levels of *A. cantonensis*. *Parmarion martensi* is an invasive species found in the Hawaiian Islands only on the islands of Oahu and Hawaii and is the species most frequently implicated in transmission of the parasite to humans in the Islands [89]. Predatory behavior of some snails, such as *A. fulica* which feed on slugs [90] and *P. canaliculata* observed to prey on other snails [91] may lead to infection in these snails via an alternative pathway other than feces.

Although the two caenogastropod species, *P. canaliculata* and *Cyclotropis* sp., had the lowest average parasite concentrations, this was based on only one positive individual for each species. These low parasite loads in *P. canaliculata* may again be due to the lower chance of this species ingesting larvae in a stream or pond than on land. Even though *A. fulica* is a relatively large (heavy) species, it harbored on average as many or fewer parasites than species only 15–25% of its mass, and one *A. fulica* had the lowest individual parasite concentration. This could be due to the muscular foot tissue being relatively poorly oxygenated and thus less preferred by *A. cantonensis* [49]. Despite *Tornatellides* sp. having a high parasite load per mg of tissue, even if a whole specimen (ca 2 mm in length) were consumed only ca 2,000 larvae (an overestimate due to the added weight of its shell, which was not removed for this extrapolation) would be ingested, while consuming a whole *L. alte* could result in ingestion of up to a thousand times more larvae.

A diverse assemblage of gastropods can thus serve as hosts of *A. cantonensis* to varying degrees. In the future, monitoring and quarantine efforts should take this into account. In Hawaii there has been a distinct focus on *P. martensi* [28], [52], [50], [55], [89], and although this species is definitely one of the most important hosts in Hawaii, it is by no means the only one. A number of species commonly found in highly populated areas at lower elevations were positive for *A. cantonensis*. Species such as *A. fulica*, *E. rosea*, *L. alte*, *P. achatinaceum*, *S. octona* and *V. cubensis* are commonly found in Hawaii in nurseries, farms and home gardens, where contact with produce can be a regular occurrence, and inadvertent consumption of infected hosts on produce is thought to be a major pathway of infection [29]. Also it is difficult to remove snails from produce [92].

Both tropical and temperate snails can carry *A. cantonensis*, indicating the potential for future expansion of the parasite's range under climate change and the need for continued concern about angiostrongyliasis as an emerging infectious disease. Since many species are potential hosts, it is likely that abiotic factors, particularly temperature and perhaps humidity, have a greater influence on infection rates and continued range expansion of the parasite than does the spread of particular host species. Knowledge of the possible vectors of *A. cantonensis* and their parasite loads is important for public health management.

## Supporting Information

Appendix S1 **Known gastropod hosts of** ***Angiostrongylus cantonensis*** , associated localities and corresponding key references.* References reporting only experimental laboratory infection - no locality is given for such studies. ** References reporting both natural and experimental infection.
(DOCX)
