# Correction: Diverse Gastropod Hosts of *Angiostrongylus cantonensis*, the Rat Lungworm, Globally and with a Focus on the Hawaiian Islands

**DOI:** 10.1371/journal.pone.0193556

**Published:** 2018-02-22

**Authors:** Jaynee R. Kim, Kenneth A. Hayes, Norine W. Yeung, Robert H. Cowie

In the Molecular detection of *A*. *cantonensis* subsection of the Materials and Methods section, there are errors in the fourth sentence of the second paragraph. The correct sentence is: Amplification parameters were 95°C for 3 min, 58°C for 1 min, and elongation at 72°C for 1 min followed by 34 cycles of 95°C for 20 s, 60°C for 20 s, and 72°C for 35 sec with a final elongation at 72°C for 10 min.
