# Supplementary material for: Diverse Gastropod Hosts of Angiostrongylus cantonensis, the Rat Lungworm, Globally and with a Focus on the Hawaiian Islands
Source: PLoS One. 2014 May 2;9(5):e94969. doi: 10.1371/journal.pone.0094969 (PMC4008484; doi:10.1371/journal.pone.0094969)
Supplement: Appendix S1 — Known gastropod hosts of Angiostrongylus cantonensis , associated localities and corresponding key references.* References reporting only experimental laboratory infection - no locality is given for such studies. ** References reporting both natural and experimental infection. (DOCX) [file pone.0094969.s001.docx]

**Appendix S1. Known gastropod hosts of *Angiostrongylus cantonensis*, associated localities and corresponding key references.**

| **Species** | **Locality** | **References** |
| --- | --- | --- |
| **Heterobranchia** |  |  |
| **Terrestrial** |  |  |
| **Achatinellidae** |  |  |
| *Lamellidea pusilla* (Gould, 1847) |  | Wallace and Rosen 1969c* |
| *Tornatellides* sp. | Hawaii | this study |
| **Achatinidae** |  |  |
| *Achatina fulica* Bowdich, 1822 | Amami Islands (Japan), Brazil, China, Florida, Hawaii, Indonesia, Malaysia, Mariana Islands, Micronesia, Ogasawara Islands (Japan), Okinawa (Japan), Papua New Guinea, Taiwan, Thailand | Alicata 1965a; Lim and Heyneman 1965; Crook et al. 1968; Wallace and Rosen 1969b; Intermill et al. 1972; Margono and Ilahude 1974; Yii et al. 1975; Brockelman et al. 1976*; Sato et al. 1980; Scrimgeour and Welch 1984; Li et al. 2006; Caldeira et al. 2007; Tokiwa et al. 2012; Teem et al. 2013; this study |
| **Agriolimacidae** |  |  |
| *Deroceras laeve* (Müller, 1774) | China, Cook Islands, Hawaii, New Caledonia, Okinawa (Japan), Tahiti | Alicata and McCarthy 1964; Wallace and Rosen 1969b, c*; Intermill et al. 1972; Ash 1976; Li et al. 2006 (as ‘*leave*’) |
| *Deroceras reticulatum* (Müller, 1774) | Hawaii | Weinstein et al. 1963*; this study |
| **Ariophantidae** |  |  |
| *Ariophantidae* sp. | China | Lv et al. 2008 |
| *Cryptosoma imperator* (Gould, 1859) |  | Ko 1991* |
| *Cryptozona bistrialis* (Beck, 1837) | Unspecified | Cross and Chen 2007 (as ‘*bristalis*’) |
| *Hemiplecta distincta* (Pfeiffer, 1850) | Thailand | Crook et al. 1968 |
| *Hemiplecta siamensis* (Pfeiffer, 1856) | Thailand | Crook et al. 1968 |
| *Macrochlamys loana* (Gredler, 1882) | China | Li et al. 2006 (unverified species name) |
| *Macrochlamys nitidissima* (Moellendorff, 1883) |  | Ko 1991* |
| *Macrochlamys resplendens* (Philippi, 1864) | Malaysia, Thailand | Liat et al. 1965; Crook et al. 1968 (as ‘*Sarika*’) |
| *Microparmarion malayanus* (Collinge, 1903) | Malaysia | Liat et al. 1965 |
| *Parmarion martensi* Simroth, 1893 | Hawaii, Okinawa (Japan) | Asato et al. 2004; Hollingsworth et al. 2007; this study |
| **Bradybaenidae** |  |  |
| *Acusta despecta* (Sowerby, 1839) | Okinawa (Japan) | Asato et al. 2004 |
| *Bradybaena brevispira* (H. Adams, 1870) | China | Lv et al. 2008 |
| *Bradybaena circulus* (Pfeiffer,1846) | Japan, Okinawa (Japan) | Intermill et al. 1972; Noda et al. 1987** |
| *Bradybaena ravida* (Benson, 1842) | China | Li et al. 2006 (‘*revida*’) |
| *Bradybaena similaris* (Rang, 1831) | Brazil, China, Cook Islands, Cuba, Hawaii, Mariana Islands, Micronesia, New Caledonia, Tahiti | Alicata 1965a; Wallace and Rosen 1969b, c*; Ash 1976; Li et al. 2006 (as ‘*similaris similaris*’); Caldeira et al. 2007; Dorta-Contreras et al. 2007 |
| *Fruticicola despecta* (A. Adams, 1868) | Okinawa (Japan) | Intermill et al. 1972; Noda et al. 1987* |
| *Plectotropis applanata* (Möellendorff, 1884) | China | Li et al. 2006 (‘*appanata*’) |
| **Camaenidae** |  |  |
| *Camaena cicatricosa* (Müller, 1774) | China | Ko 1991 (‘*circatoricosa*’)*; Lv et al. 2008 |
| *Satsuma mercatoria* (Pfeiffer, 1845) | Okinawa (Japan) | Intermill et al. 1972 |
| *Trichochloritis hungerfordianus* (Möllendorff, 1884) | China | Lv et al. 2008 |
| *Trichochloritis rufopila* (Möllendorff, 1884) | China | Lv et al. 2008 |
| **Cepolidae** |  |  |
| *Jeanneretia bicincta* (Menke, 1830) | Cuba | Dorta-Contreras et al. 2007 |
| **Discidae** |  |  |
| *Anguispira alternata* (Say, 1816) |  | Campbell and Little 1988* |
| **Dyakiidae** |  |  |
| *Quantula striata* (Gray, 1834) | Malaysia | Lim and Heyneman 1965 |
| **Enidae** |  |  |
| *Luchuena reticulata* (Reeve, 1849) |  | Noda et al. 1987* |
| **Euconulidae** |  |  |
| *Liardetia sculpta* (Möllendorff, 1883) |  | Wallace and Rosen 1969c* |
| **Helicarionidae** |  |  |
| *Girasia peguensis* (Theobald, 1864) | Malaysia | Liat et al. 1965 |
| *Helicarion* sp. |  | Yong et al. 1981* |
| *Ovachlamys fulgens* (Gude, 1900) | Hawaii | Qvarnstrom et al. 2013; this study |
| *Philonesia* sp. | Hawaii | this study |
| **Helicidae** |  |  |
| *Cornu aspersum* (Müller, 1774) |  | Ash 1976 (as ‘*Helix aspersa*’)* |
| *Emoda sagraiana* (D'Orbigny, 1842) | Cuba | Dorta-Contreras et al. 2007 (as ‘*sagrayana*’) |
| *Helicina adspersa* Pfeiffer, 1839 | Cuba | Dorta-Contreras et al. 2007 |
| *Helicina orbiculata* (Say, 1818) |  | Alicata 1965b* |
| *Viana regina* (Morelet, 1849) | Cuba | Dorta-Contreras et al. 2007 |
| **Helminthoglyptidae** |  |  |
| *Polymita picta* (Born, 1780) | Cuba | Dorta-Contreras et al. 2007 |
| **Limacidae** |  |  |
| *Lehmannia poirieri* (Mabille, 1883) |  | Campbell and Little 1988* |
| *Lehmannia valentiana* (Férussac, 1822) | Hawaii, Okinawa (Japan) | Asato et al. 2004 (as ‘*Limax valentianus*’); this study |
| *Limax flavus* Linnaeus, 1758 | China, Okinawa (Japan) | Sato et al. 1981; Campbell and Little 1988*; Li et al. 2006 (‘*Zimax*’) |
| *Limax marginatus* (Müller, 1774) | Japan | Tokiwa et al. 2012 |
| *Limax maximus* Linnaeus, 1758 | China, Hawaii | Weinstein et al. 1963*; Yang et al. 2012; this study |
| **Oleacinidae** |  |  |
| *Oleacina solidula* (Pfeiffer, 1840) | Cuba | Dorta-Contreras et al. 2007 |
| **Oxychilidae** |  |  |
| *Oxychilus alliarius* (Miller, 1822) | Hawaii | this study |
| **Philomycidae** |  |  |
| *Meghimatium bilineatum* (Benson, 1842) | China, Okinawa (Japan) | Sato et al. 1981 (as ‘*Incilaria bilineata*’); Li et al. 2006 (as ‘*bilinestum*’) |
| *Pallifera* sp. | Hawaii | Qvarnstrom et al. 2013 |
| *Philomycus bilineatus* (Benson, 1842) | China, Okinawa (Japan) | Intermill et al. 1972; Lv et al. 2008 (‘*Phiolomycus*’) |
| *Philomycus carolinianus* (Bosc, 1801) |  | Campbell and Little 1988* |
| **Pleurodontidae** |  |  |
| *Pleurodonte* sp. | Jamaica | Robinson et al. 2013 |
| *Thelidomus aspera* (Férussac, 1821) | Jamaica | Lindo et al. 2002 (as ‘*asper*’) |
| *Zachrysia auricoma* (Férussac, 1821) | Cuba | del Risco Barrios and Diéguez 2004 |
| **Polygyridae** |  |  |
| *Mesodon thyroidus* (Say, 1817) |  | Campbell and Little 1988* |
| *Polygyra triodontoides* (Bland, 1861)* |  | Campbell and Little 1988* |
| **Sagdidae** |  |  |
| *Aquebana belutina* (Lamarck, 1822) | Puerto Rico | Andersen et al. 1986 |
| *Sagda* sp. | Jamaica | Robinson et al. 2013 |
| **Spiraxidae** |  |  |
| *Euglandina rosea* (Férussac, 1821) | Hawaii | Wallace and Rosen 1969b; Campbell and Little 1988*; this study |
| **Subulinidae** |  |  |
| *Allopeas gracile* (Hutton, 1834) |  | Wallace and Rosen 1969c* (as ‘*Lamellaxis oparanum*’) |
| *Opeas javanicum* | Mariana Islands, Micronesia | Alicata 1965a |
| *Paropeas achatinaceum* (Pfeiffer, 1846) | Cook Islands, Hawaii, Tahiti | Wallace and Rosen 1969b, c* (as ‘*Prosopeas javanicum*’); this study |
| *Rumina decollata* (Linnaeus, 1758) | Cuba | Dorta-Contreras et al. 2007 |
| *Subulina octona* (Bruguière, 1792) | Brazil, Cook Islands, Cuba, Hawaii, Mariana Islands, Micronesia, Puerto Rico, Tahiti | Alicata 1965a; Wallace and Rosen 1969b, c*; Andersen et al. 1986; del Risco Barrios and Diéguez 2004; Caldeira et al. 2007; this study |
| **Succineidae** |  |  |
| *Succinea* sp. | Cuba | del Risco Barrios and Diéguez 2004 |
| **Urocoptidae** |  |  |
| *Tetrentodon* (*Cilindricoptis*) sp. | Cuba | Dorta-Contreras et al. 2007 |
| *Tetrentodon* (*Scalaricoptis*) *filiola* (Jaume and Torre, 1972) | Cuba | Dorta-Contreras et al. 2007 |
| *Tetrentodon* (*Tetrentodon*) *perdidoensis* (Jaume and Torre, 1972) | Cuba | Dorta-Contreras et al. 2007 |
| **Veronicellidae** |  |  |
| *Laevicaulis alte* (Férussac, 1822) | Australia, China, Fiji, Hawaii, India, Indonesia, Malaysia, New Caledonia, Okinawa (Japan), Philippines | Liat et al. 1965; Wallace and Rosen 1969b, c*; Margono and Ilahude 1974; Ash 1976; Mason et al. 1976; Sato et al. 1981; Renapurkar et al. 1982; Uchikawa et al. 1984; Li et al. 2006; Fontanilla and Wade 2008 (as ‘*altae*’); this study |
| *Sarasinula linguaeformis* (Semper, 1885) | Brazil | Espírito-Santo et al. 2013 |
| *Sarasinula marginata* (Semper, 1885) | Brazil | Caldeira et al. 2007 |
| *Sarasinula plebeia* (Fischer, 1868) | Australia, Cook Islands, Fiji, Mariana Islands, Micronesia, New Caledonia, Okinawa (Japan), Tahiti | Alicata 1962, 1965a (as ‘*Vaginalus plebeius*’); Wallace and Rosen 1969b (as ‘*Vaginulus plebeius*’); Intermill et al. 1972 (as ‘*Vaginulus plebeius*’); Mason et al. 1976 |
| *Vaginulus* *ameghini* (Gambetta, 1923) |  | Campbell and Little 1988* |
| *Vaginulus* sp. | China | Lv et al. 2008 |
| *Vaginulus yuxjsjs* | China | Lv et al. 2008 (as ‘sp. nov.’ but possibly never described) |
| *Veronicella cubensis* (Pfeiffer, 1840) | Cuba, Hawaii | Qvarnstrom et al. 2007; Dorta-Contreras et al. 2007; this study |
| *Veronicella siamensis* (Martens, 1867) | Thailand | Crook et al. 1968 |
| Veronicellidae spp. | Jamaica | Robinson et al. 2013 |
| **Vertiginidae** |  |  |
| *Gastrocopta pediculus* (Shuttleworth, 1852) |  | Wallace and Rosen 1969c* |
| **Freshwater** |  |  |
| **Lymnaeidae** |  |  |
| *Fossaria viridis* (Quoy and Gaimard, 1832) |  | Alicata and Brown 1962 (as ‘*ollula*’)* |
| *Lymnaea columella* Say, 1817 |  | Kocan 1972* |
| *Lymnaea japonica* (Jay, 1856) |  | Shiota et al. 1980* |
| *Lymnaea palustris* (Müller, 1774) |  | Kocan 1972* |
| *Lymnaea* spp. |  | el-Shazly et al. 2002 |
| *Lymnaea stagnalis* (Linnaeus, 1758) |  | Kocan 1972* |
| *Lymnaea swinhoei* H. Adams, 1866 |  | Chang et al. 1968* (as ‘*swinhoe*’) |
| *Lymnaea tomentosa* (Pfeiffer, 1855) | Unspecified | Morley 2010 |
| *Lymnaea volutata* Gould, 1848 |  | Wallace and Rosen 1969c* |
| *Radix natalensis* (Krauss, 1848) | Egypt | Ibrahim 2007 (as ‘*Lymnaea*’) |
| *Stagnicola elodes* |  | Morley 2010* |
| *Stagnicola emarginata* (Say, 1821) |  | Kocan 1972* |
| **Physidae** |  |  |
| *Physa acuta* Draparnaud, 1805 |  | Shiota et al. 1980* |
| *Physa elliptica* Lea, 1843 |  | Wallace and Rosen 1969c* |
| *Physa* spp. |  | Richards and Merritt 1967* |
| *Physastra* sp. |  | Yong et al. 1981* |
| **Planorbidae** |  |  |
| *Biomphalaria alexandrina* (Ehrenberg, 1831) | Egypt | Ibrahim 2007 |
| *Biomphalaria glabrata* (Say, 1818) |  | Richards and Merritt 1967*; el-Shazly et al. 2002 |
| *Biomphalaria heliophila* (Orbigny, 1835) |  | Richards and Merritt 1967* |
| *Biomphalaria obstructa* (Morelet, 1849) |  | Richards and Merritt 1967* |
| *Biomphalaria pallida* (Adams, 1846) |  | Richards and Merritt 1967* |
| *Biomphalaria pfeifferi* (Krauss, 1848) |  | Richards and Merritt 1967* |
| *Biomphalaria straminea* (Dunker, 1848) |  | Richards and Merritt 1967* |
| *Biomphalaria tenagophila* (Orbigny, 1835) |  | Richards and Merritt 1967* |
| *Bulinus africanus* (Krauss, 1848) |  | Morley 2010* |
| *Bulinus contortus* (Michaud, 1829) |  | Morley 2010* |
| *Bulinus forskalii* (Ehrenberg, 1831) |  | Richards and Merritt 1967* |
| *Bulinus globosus* (Morelet, 1866) |  | Richards and Merritt 1967* |
| *Bulinus senegalensis* (Müller, 1781) |  | Richards and Merritt 1967* |
| *Bulinus tropicus* (Krauss, 1848) |  | Richards and Merritt 1967* |
| *Bulinus truncatus* (Audouin, 1827) | Egypt | Richards and Merritt 1967*; el-Shazly et al. 2002 |
| *Drepanotrema simmonsi* (Ferguson and Gerhardt, 1956) |  | Richards and Merritt 1967* |
| *Ferrissia tenuis* (Bourguignat, 1862) |  | Richards and Merritt 1967* |
| *Gyraulus hiemantium* (Westerlund, 1887) |  | Shiota et al. 1980* |
| *Helisoma* sp. |  | Richards and Merritt 1967* |
| *Indoplanorbis exustus* (Deshayes, 1834) | Malaysia | Liat et al. 1965; Richards and Merritt 1967* |
| *Planorbella duryi* Pilsbry, 1934 |  | Wallace and Rosen 1969c* (as ‘*Helisoma duryi normale*’) |
| *Plesiophysa hubendicki* Richards and Ferguson, 1962 |  | Richards and Merritt 1967* |
| *Segmentina hemisphaerula* (Benson, 1842) |  | Chang et al. 1968* |
|  |  |  |
| **Caenogastropoda** |  |  |
| **Terrestrial** |  |  |
| **Assimineidae** |  |  |
| *Assiminea parvula* (Pease, 1865) |  | Wallace and Rosen 1969c (as ‘*nitida*’)* |
| *Cyclotropis* sp. | Hawaii | this study |
| *Omphalotropis fragilis* Pease, 1860 |  | Wallace and Rosen 1969c (as ‘*Omphalatropis*’)* |
| **Cyclophoridae** |  |  |
| *Chamalycaeus sinensis* (Heude, 1882) |  | Ko 1991 (as ‘Cyclotus’)* |
| **Megalomastomatidae** |  |  |
| *Farcimen tortum* (Wood, 1828) | Cuba | Dorta-Contreras et al. 2007 |
| **Neocyclotidae** |  |  |
| *Poteria* sp. | Jamaica | Robinson et al. 2013 |
| **Pomatiidae** |  |  |
| *Chondropoma pictum arangoi* (Torre and Bartsch, 1938) | Cuba | Dorta-Contreras et al. 2007 |
| *Eutudora jimenoi* (Arango in Pfeiffer, 1864) | Cuba | Dorta-Contreras et al. 2007 |
| *Rhitidopoma* sp. | Cuba | Dorta-Contreras et al. 2007 |
| **Pupinidae** |  |  |
| *Pupina complanata* (Pease, 1860) | Micronesia | Alicata 1965a |
| **Truncatellidae** |  |  |
| *Truncatella marginata* Küster, 1855 |  | Wallace and Rosen 1969c* |
| **Freshwater** |  |  |
| **Ampullariidae** |  |  |
| *Lanistes carinatus* (Olivier, 1804) | Egypt | Yousif and Ibrahim 1978 |
| *Marisa cornuarietis* (Linnaeus, 1758)* |  | Richards and Merritt 1967* |
| *Pila ampullacea* (Linnaeus, 1758) | Thailand | Punyagupta 1965 |
| *Pila angelica* (Annandale, 1920)* |  | Morley 2010* |
| *Pila gracilis* (Lea, 1856) | Thailand | Crook et al. 1968 |
| *Pila pesmei* (Morlet, 1889) | Thailand | Tesana et al. 2009 |
| *Pila scutata* (Mousson, 1849) | Indonesia, Malaysia, Thailand | Lim and Heyneman 1965; Punyagupta et al. 1970; Margono and Ilahude 1974 |
| *Pila turbinis* Lea, 1856 | Thailand | Crook et al. 1968 (as ‘*Pila ampullacea turbinis*’) |
| *Pila virescens* (Deshayes, 1830) (as ‘*polita*’) | China, Thailand | Crook et al. 1968; Tesana et al. 2008*; Cheng et al. 2011 |
| *Pomacea canaliculata* (Lamarck, 1822) | China, Hawaii, Okinawa (Japan), Taiwan | Nishimura et al. 1986 (as ‘*Ampullarius canaliculatus*’)**; Yen et al. 1990 (as ‘*Ampullarium canaliculatus*’); Zhang et al. 2008; this study |
| *Pomacea lineata* (Spix in Wagner, 1827) | Brazil | Thiengo et al. 2010 |
| *Pomacea maculata* Perry, 1810 | China, Louisiana | Li et al. 2006 (as ‘*Pila gigas*’); Li et al. 2012 (as ‘*Ampullarum crossean*’)*; Teem et al. 2013 |
| *Pomacea paludosa* (Say, 1829) | Cuba, Hawaii | Wallace and Rosen 1969b, c*; del Risco Barrios and Diéguez 2004 |
| **Bithyniidae** |  |  |
| *Bithynia siamensis goniomphalos* (Lea, 1856) | Thailand | Tesana et al. 2009 |
| **Buccinidae** |  |  |
| *Clea helena* (Philippi, 1847) | Thailand | Tesana et al. 2009 |
| **Paludomidae** |  |  |
| *Cleopatra bulimoides* (Olivier, 1804) | Egypt | Ibrahim 2007 |
| *Cleopatra cyclostomoides* (Bourguignat, 1879) | Egypt | Ibrahim 2007 |
| **Pleuroceridae** |  |  |
| *Goniobasis livescens* (Menke, 1830) |  | Kocan 1972* |
| **Thiaridae** |  |  |
| *Melanoides tuberculata* (Müller, 1774) | Egypt, Thailand | Crook et al. 1968; Ibrahim 2007 |
| **Viviparidae** |  |  |
| *Bellamya aeruginosa* (Reeve, 1862) | China | Li et al. 2006 |
| *Bellamya chinensis* (Gray, 1834) | Taiwan | Chang et al. 1968 (as ‘*Cipangopaludina*’); Ko 1991 (as ‘*Cipangopalaudina*’)* |
| *Bellamya lithophaga* (Heude, 1889) | China | Cheng et al. 2011 |
| *Bellamya quadrata* (Benson, 1842) | China | Chang et al. 1968 (as ‘*Sinotaia*’)*; Xie and Wu 2013 (as ‘*Sinotaia*’) |
| *Bellamya* spp. | China | Li et al. 2006 |
| *Cipangopaludina* sp. | China | Deng et al. 2012 |
| *Filopaludina martensi* (Lea, 1856) | Malaysia | Liat et al. 1965 (as ‘*Bellamya ingallsiana*’) |
| *Filopaludina martensi martensi* (Frauenfeld, 1865) | Thailand | Tesana et al. 2009 |
| *Filopaludina sumartrensis polygramma* (Dunker, 1852) | Thailand | Tesana et al. 2009 |
| *Sinotaia histrica* (Gould, 1859) |  | Shiota et al. 1980 (as ‘*Taia*’)* |
| *Sinotaia martensiana* (Nevill, 1881) | Unspecified | Morley 2010 |
| **Marine** |  |  |
| **Architectonicidae** |  |  |
| *Discotectonica acutissima* (Sowerby, 1914) | China | Yang et al. 2012 (doubtful record) |
| **Species of uncertain identity** |  |  |
| *P. bilineatus* | China | Li et al. 2006 |

* References reporting only experimental laboratory infection - no locality is given for such studies.

** References reporting both natural and experimental infection.

**References**

1. Alicata JE (1962) Observations on the occurrence of the rat-lungworm *Angiostrongylus cantonensis* in New Caledonia and Fiji. J Parasitol 48: 595.

2. Alicata JE (1965a) Notes and observations on murine angiostrongylosis and eosinophilic meningoencephalitis in Micronesia. Can J Zool 43: 667-672.

3. Alicata JE (1965b) Biology and distribution of the rat lungworm, *Angiostrongylus cantonensis*, and its relationship to eosinophilic meningoencephalitis and other neurological disorders of man and animals. Adv Parasitol 3: 223-248.

4. Alicata JE, Brown RW (1962) Observations on the method of human infection with *Angiostrongylus cantonemis* in Tahiti. Can J Zool 40: 755-760.

5. Alicata JE, McCarthy DD (1964) On the incidence and distribution of the rat lungworm *Angiostrongylus cantonensis* in the Cook Islands, with observations made in New Zealand and Western Samoa. Can J Zool 42: 605-611.

6. Andersen E, Gubler DJ, Sorensen K, Beddard J, Ash LR (1986) First report of *Angiostrongylus cantonensis* in Puerto Rico. Am J Trop Med Hyg 35: 319-322.

7. Asato R, Taira K, Nakamura M, Kudaka J, Itokazu K, et al. (2004) Changing epidemiology of angiostrongyliasis cantonensis in Okinawa Prefecture, Japan. Jpn J Infect Dis 57: 184-186.

8. Ash LR (1976) Observations on the role of mollusks and planarians in the transmission of *Angiostrongylus cantonensis* infection to man in New Caledonia. Rev Biol Trop 24: 163-174.

9. Brockelman CR, Chusatayanond W, Baidikul V (1976) Growth and localization of *Angiostrongylus cantonensis* in the molluscan host, *Achatina fulica*. Southeast Asian J Trop Med Public Health 7: 30-37.

10. Caldeira RL, Mendonça CLGF, Goveia CO, Lenzi HL, Graeff-Teixeira C, et al. (2007) First record of mollusks naturally infected with *Angiostrongylus cantonensis* (Chen, 1935) (Nematoda: Metastrongylidae) in Brazil. Mem Inst Oswaldo Cruz 102: 887-889.

11. Campbell BG, Little MD (1988) The findings of *Angiostrongylus cantonensis* in rats in New Orleans. Am J Trop Med Hyg 38: 568-573.

12. Chang P-K, Cross JH, Chen SSS (1968) Aquatic snails as intermediate hosts for *Angiostrongylus cantonensis* on Taiwan. J Parasitol 54: 183-183.

13. Cheng YZ, Hou J, He XH, Hong ZK, Li LS, et al. (2011) Prevalence of *Paragonimus* and *Angiostrongylus cantonensis* infections in snails in southeastern China. J Anim Vet Adv 10: 2599-2602.

14. Crook JR, Fulton SE, Supanwong K (1968) Ecological studies on the intermediate and definitive hosts of *Angiostrongylus cantonensis* (Chen, 1935) in Thailand. Ann Trop Med Parasitol 62: 27-44.

15. Cross JH, Chen ER (2007) Angiostrongyliasis. In: Murrell KD, Fried B, editors. Food-borne parasitic zoonoses: fish and plant-borne parasites. New York: Springer Science + Business Media, LLC. pp. 263-290.

16. del Risco Barrios U, Diéguez L (2004) Presencia y distribución de hospederos intermediarios de *Angiostrongylus cantonensis* en Camagüey. Prevalencia e importancia epidemiológica para su control. Archivo Médico de Camagüey 8: 1025-0255.

17. Deng Z-H, Zhang Q-M, Huang S-Y, Jones JL (2012) First provincial survey of *Angiostrongylus cantonensis* in Guangdong Province, China. Trop Med Int Health 17: 119-122.

18. Dorta-Contreras AJ, Núñez-Fernández FA, Pérez-Martin O, Lastre-González M, Magraner-Tarrau ME, et al. (2007) Peculiaridades de la meningoencefalitis por *Angiostrongylus cantonensis* en América. Rev Neurol 45: 755-763.

19. el-Shazly AM, el-Hamshary EM, el-Shewy KM, Rifaat MM, el-Sharkawy IM (2002) Incidence of *Parastrongylus cantonensis* larvae in different freshwater snails in Dakahlia Governorate. J Egypt Soc Parasitol 32: 579-588.

20. Espírito-Santo MCC do, Pinto PLS, da Mota DJG, Gryschek RCB (2013) The first case of *Angiostrongylus cantonensis* eosinophilic meningitis diagnosed in the city of São Paulo, Brazil. Rev I Med Trop 55: 129-132.

21. Fontanilla IKC, Wade CM (2008) The small subunit (SSU) ribosomal (r) RNA gene as a genetic marker for identifying infective 3^rd^ juvenile stage *Angiostrongylus cantonensis*. Acta Trop 105: 181-186.

22. Hollingsworth RG, Kaneta R, Sullivan JJ, Bishop HS, Qvarnstrom Y, et al. (2007) Distribution of *Parmarion cf. martensi* (Pulmonata: Helicarionidae), a new semi-slug pest on Hawaii Island, and its potential as a vector for human angiostrongyliasis. Pac Sci 61: 457-467.

23. Ibrahim MM (2007) Prevalence and intensity of *Angiostrongylus cantonensis* in freshwater snails in relation to some ecological and biological factors. *Parasite* 14: 61-70.

24. Intermill RW, Palmer CP, Fredrick RM, Tamashiro H (1972) *Angiostrongylus cantonensis* on Okinawa. Jpn J Exp Med 42: 355-359.

25. Ko RC (1991) Current status of food-borne parasitic zoonoses in Hong Kong. Southeast Asian J Trop Med Public Health 22 (Supplement): 42-47.

26. Kocan AA (1972) Some common North American aquatic snails as experimental hosts of *Angiostrongylus cantonensis*: with special reference to *Lymnaea palustris*. J Parasitol 58: 186-187.

27. Li L-S, Zhou Z-N, Lin J-X, Zhang Y, Cheng Y-Z, et al. (2006) Discovery of six species of new hosts for *Angiostrongylus cantonensis* and investigation of the epidemic foci in Fujian province. Zhongguo Ren Shou Gong Huan Bing Za Zhi 22: 533-537.

28. Li Z-Y, Sun R, Li J, Song Y-X, Lin Y-C, et al. (2012) Efficacy of albendazole combined with a marine fungal extract (m2-9) against *Angiostrongylus cantonensis*-induced meningitis in mice. J Helminth 86: 410-417.

29. Liat LB, Kong O-YC, Joe LK (1965) Natural infection of *Angiostrongylus cantonensis* in Malaysian rodents and intermediate hosts, and preliminary observations on acquired resistance. Am J Trop Med Hyg 14: 610-617.

30. Lim BL, Heyneman D (1965) Host-parasite studies of *Angiostrongylus cantonensis* (Nematoda, Metastrongylidae) in Malaysaian rodents: natural infection of rodents and molluscs in urban and rural areas of central Malaya. Ann Trop Med Parasitol 59: 425-433.

31. Lindo JF, Waugh C, Hall J, Cunningham-Myrie C, Ashley D, et al. (2002) Enzootic *Angiostrongylus cantonensis* in rats and snails after an outbreak of human eosinophilic meningitis, Jamaica. Emerg Infect Dis 8: 324-326.

32. Lv S, Zhang Y, Steinmann P, Zhou X-N (2008) Emerging angiostrongyliasis in mainland China. Emerg Infect Dis 14: 161-164.

33. Margono SS, Ilahude HD (1974) *Angiostrongylus cantonensis* in rats and intermediate hosts in Jakarta and its vicinity. Southeast Asian J Trop Med Public Health 5: 236-240.

34. Mason KV, Prescott CW, Kelly WR, Waddell AH (1976) Granulomatous encephalomyelitis of puppies due to *Angiostrongylus cantonensis*. Aust Vet J 52: 295.

35. Morley NJ (2010) Aquatic molluscs as auxiliary hosts for terrestrial nematode parasites: implications for pathogen transmission in a changing climate. Parasitol 137: 1041-1056.

36. Nishimura K, Mogi M, Okazawa T, Sato Y, Toma H, et al. (1986) *Angiostrongylus cantonensis* infection in *Ampullarius canaliculatus* (Lamarck) in Kyushu, Japan. The Southeast Asian J Trop Med Public Health 17: 595-600.

37. Noda S, Uchikawa R, Matayoshi S, Watanabe Y, Sato A (1987) Observations on the transmission of *Angiostrongylus cantonensis* from snail to rodent. J Helminthol 61: 241-246.

38. Punyagupta S (1965) Eosinophilic meningoencephalitis in Thailand: Summary of nine cases and observations on *Angiostrongylus cantonensis* as a causative agent and *Pila ampullacea* as a new intermediate host. Am J Trop Med Hyg 14: 370-374.

39. Punyagupta S, Bunnag T, Juttijudata P, Rosen L (1970) Eosinophilic meningitis in Thailand: epidemiologic studies of 484 typical cases and the etiologic role of *Angiostrongylus cantonensis*. Am J Trop Med Hyg 19: 950-958.

40. Qvarnstrom Y, Sullivan JJ, Bishop HS, Hollingsworth R, da Silva AJ (2007) PCR-based Detection of *Angiostrongylus cantonensis* in Tissue and Mucus Secretions from Molluscan Hosts. Appl Environ Microbiol 73: 1415-1419.

41. Qvarnstrom Y, Bishop HS, da Silva AJ (2013) Detection of rat lungworm in intermediate, definitive, and paratenic hosts obtained from environmental sources. Hawaii J Med Public Health 72 (Supplement 2): 63-69.

42. Renapurkar MK, Bhopale MK, Limaye LS, Sharma KD (1982) Prevalence of *Angiostrongylus cantonensis* infection in commensal rats in Bombay. J Helminthol 56: 345-349.

43. Richards CS, Merritt JW (1967) Studies on *Angiostrongylus cantonensis* in molluscan intermediate hosts. J Parasitol 53: 382-388.

44. Robinson RD, Waugh CA, Todd CD, Lorenzo-Morales J, Lindo JF (2013) Rat lungworm: an emerging zoonosis in Jamaica. Hawaii J Med Public Health 72 (Supplement 2): 33-34.

45. Sato A, Noda S, Nojima H, Yuyama Y, Kawabata N, et al. (1980) A survey of *Angiostrongylus cantonensis* in the Amami Islands. I. The occurrence of *A. cantonensis* in snails and rodents in Yoron-jima. Kisechugaku Zasshi 29: 383-391.

46. Sato Y, Otsuru M, Asato R, Yamashita T (1981) An epidemiological survey on *Angiostrongylus cantonensis* and angiostrongyliasis in the Southwest Islands, Japan. Ryukyu University Journal of Health Sciences and Medicine 4:15-26.

47. Scrimgeour EM, Welch JS (1984) *Angiostrongylus cantonensis* in East New Britain, Papua New Guinea. Trans R Soc Trop Med Hyg 78: 774-775.

48. Shiota T, Arizono N, Yamada M, Kurimoto H, Shimada Y, et al. (1980) Experimental infections of 13 species of Japanese freshwater mollusks with *Angiostrongylus cantonensis* larvae. Kisechugaku Zasshi 29: 27-38.

49. Teem JL, Qvarnstrom Y, Bishop HS, da Silva AJ, Carter J, et al. (2013) The occurrence of the rat lungworm, *Angiostrongylus cantonensis*, in nonindigenous snails in the Gulf of Mexico region of the United States. Hawaii J Med Public Health 72 (Supplement 2): 11-14.

50. Tesana S, Srisawangwong T, Sithithaworn P, Laha T (2008) *Angiostrongylus cantonensis*: experimental study on the susceptibility of apple snails *Pomacea canaliculata* compared to *Pila polita*. Exp Parasitol 118: 531-535.

51. Tesana S, Srisawangwong T, Sithithaworn P, Laha T, Andrews R (2009) Prevalence and intensity of infection with third stage larvae of *Angiostrongylus cantonensis* in mollusks from northeast Thailand. J Trop Med Hyg 80: 983-987.

52. Thiengo SC, Maldonado A, Mota EM, Torres EJL, Caldeira R, et al. (2010) The giant African snail *Achatina fulica* as natural intermediate host of *Angiostrongylus cantonensis* in Pernambuco, northeast Brazil. Acta Trop 115: 194-199.

53. Tokiwa T, Harunari T, Tanikawa T, Komatsu N, Koizumi N, et al. (2012) Phylogenetic relationships of rat lungworm, *Angiostrongylus cantonensis*, isolated from different geographical regions revealed widespread multiple lineages. Parasitol Int 61: 431-436.

54. Uchikawa R, Takagi M, Matayoshi S, Sato A (1984) The presence of *Angiostrongylus cantonensis* in Viti Levu, Fiji. J Helminthol 58: 231-234.

55. Wallace GD, Rosen L (1969b) Studies on eosinophilic meningitis. V. Molluscan hosts of *Angiostrongylus cantonensis* on Pacific islands. Am J Trop Med Hyg 18: 206-216.

56. Wallace GD, Rosen L (1969c) Techniques for recovering and identifying larvae of *Angiostrongylus cantonensis* from molluscs. Malacologia 7: 427-438.

57. Weinstein PP, Rosen L, Laqueur GL, Sawyer TK (1963) *Angiostrongylus cantonensis* infection in rats and rhesus monkeys, and observations on the survival of the parasite *in vitro*. Am J Trop Med Hyg 12: 358-377.

58. Xie P, Wu D-R (2013) The survey of the natural epidemic foci of *Angiostrongylus cantonensis* in Beihai City of Guangxi. Guo Ji Yi Xue Ji Sheng Chong Bing Za Zhi 40: 67-70.

59. Yang X, Qu Z, He H, Zheng X, He A, et al. (2012) Enzootic angiostrongyliasis in Guangzhou, China, 2008-2010. Am J Trop Med Hyg 86: 846-849.

60. Yen CM, Chen ER, Cheng CW (1990) A survey of *Ampullarium canaliculatus* for natural infection of *Angiostrongylus cantonensis* in south Taiwan. J Trop Med Hyg 93: 347-350.

61. Yii C-Y, Chen C-Y, Chen E-R, Hsieh H-C, Shih C-C, et al. (1975) Epidemiologic studies of eosinophilic meningitis in southern Taiwan. Am J Trop Med Hyg 24: 447-454.

62. Yong WK, Welch JS, Dobson C (1981) Localized distribution of *A. cantonensis* among wild rat populations in Brisbane, Australia. Southeast Asian J Trop Med Public Health 12: 608-609.

63. Yousif F, Ibrahim A (1978) The first record of *Angiostrongylus cantonensis* from Egypt. Z Parasitenkd 56: 73-80.

64. Zhang R-L, Chen M-X, Gao S-T, Geng Y-J, Huang D-N, et al. (2008) Enzootic angiostrongyliasis in Shenzhen, China. Emerg Infect Dis 14: 1955-1956.
